# Personalized Medicine Using Neuroimmunological Biomarkers in Depressive Disorders

**DOI:** 10.3390/jpm11020114

**Published:** 2021-02-10

**Authors:** Suhyuk Chi, Moon-Soo Lee

**Affiliations:** Department of Psychiatry, Korea University Guro Hospital, Seoul 08308, Korea; link0710@hotmail.com

**Keywords:** cytokines, neuroimmunology, depression, machine learning

## Abstract

Major depressive disorder (MDD) is associated with increased suicidal risk and reduced productivity at work. Neuroimmunology, the study of the immune system and nervous system, provides further insight into the pathogenesis and outcome of MDD. Cytokines are the main modulators of neuroimmunology, and their levels are somewhat entangled in depressive disorders as they affect depressive symptoms and are affected by antidepressant treatment. The use of cytokine-derived medication as a treatment option for MDD is currently a topic of interest. Although not very promising, cytokines are also considered as possible prognostic or diagnostic markers for depression. The machine learning approach is a powerful tool for pattern recognition and has been used in psychiatry for finding useful patterns in data that have translational meaning and can be incorporated in daily clinical practice. This review focuses on the current knowledge of neuroimmunology and depression and the possible use of machine learning to widen our understanding of the topic.

## 1. Introduction

Major depressive disorder (MDD) is one of the leading causes of disability in the present world, creating high temporal and financial costs. It not only increases suicidal risks but also contributes to higher risk of cardiovascular death [1]. Reduced workplace productivity is another outcome of depression, leading to an estimated loss of $36.6 billion per year in the United States [2]. This provides a major reason for researchers to investigate the etiopathogenesis of depression and search for various approaches for the amelioration of its symptoms. Many theories attempt to explain the origin and development of symptoms of depression. Recently accumulated evidence suggests that the immune system might be associated with psychiatric disorders [3]. This idea has been transformed into a scientific field that is now called neuroimmunology.

Neuroimmunology is the study of the effect of the immune system on the nervous system and vice versa. Although it was previously considered that the blood-brain barrier (BBB) effectively segregates the brain from the bloodstream and its many circulating elements, it is now clear that the brain is not a secluded area. Immunologic modulators, namely, cytokines, affect the central nervous system and may control both behavior and emotions [4]. 

Cytokines are small proteins involved in various interactions as immuno-modulators. Most of these were previously categorized as interleukins (IL), lymphokines, interferons (IFN), chemokines, or tumor necrosis factors (TNF), as they were considered to be only released by certain types of cells. It is now known that one specific cytokine can be produced by multiple cell types, thus, the collective term “cytokine” is used. These act as signaling molecules that mediate both cellular and humoral immunity.

Hard evidence suggests that cytokines have certain effects on various psychiatric disorders including schizophrenia, depression, and Alzheimer’s disease [5,6]. In this article, we review our current knowledge on the role of cytokines in depression and the possible implications of machine learning (ML) in the current and future research in this field.

Although artificial intelligence (AI) has been in use in the field of neuroscience, particularly depression, the clinical application of AI has expanded in recent years. Although the number of studies on this are increasing, there are limited reviews about the application of AI in the diagnosis of depression. This year, the number of studies on the clinical application of AI doubled compared to that in the previous year. The number of studies conducted and cited in 2018 makes it an important year for AI research [7]. ML, a tool for automated analysis, involves learning from previous datasets, which helps to make clinical decisions based on new datasets without explicit algorithms. Two areas that may benefit from the application of ML techniques in the medical field are diagnosis and outcome prediction [8]. As we narrowed the scope of the paper to the use of biochemical data including neuroimmunological biomarkers for depression and ML as a methodology of AI, we found that the number of such studies were limited; therefore, we also discuss the current research findings on the general use of ML in depressive disorders in this review.

## 2. General Considerations for Neuroimmunology in Depression

### 2.1. Cytokines and Depression

Cytokines can be divided into pro-inflammatory and anti-inflammatory, depending on their main role. These categories might not fit in the field of psychiatry since their lack of nuance can oversimplify the delicate actions of various cytokines, especially in regard to depressive disorders. Table 1 shows a very brief pre-emptive summary of the role of cytokines in depression.

Smith was an early proponent of the possibility of cytokines affecting depression with “the macrophage theory of depression” [24]. It was proposed that excessive secretion of monokines could cause depressive symptoms in volunteers, further suggesting that eicosapentaenoic acids might act protectively against depression by suppressing macrophages. This idea was further developed by Maes et al., who proposed that the inflammatory response system influences the hypothalamic-pituitary-adrenal (HPA) axis, thus, influencing serotonin and catecholaminergic turnover and inducing depressive symptoms [25].

A more recent meta-analysis of 24 studies reported that IL-6 and TNF-α showed significantly higher concentrations in depressed patients than in controls [5]. IL-6 is mainly secreted by macrophages and monocytes, stimulating the differentiation and proliferation of B-cells, whereas TNF-α is secreted by macrophages, mast cells, and natural killer cells, further stimulating the secretion of other cytokines [26,27]. TNF-α was not only found to be increased in patients with depression but was also shown to induce depressive symptoms if injected directly; such symptoms improved after administration of anti-TNF-α agents [16,17,18]. In a meta-analysis, D’Acunto et al. reported that TNF-α levels were not significantly related to depression, although it is noteworthy that the analysis was only targeted at children and adolescents [28]. Furthermore, Brambilla et al. reported decreased levels of TNF-α in patients with suicidality or dysthymia [29]. In addition to TNF-α, other cytokines also induced depressive symptoms if administered directly to patients. The use of IL-2 and IFN-α, mostly to treat hepatitis or cancer, resulted in depressive symptoms such as apathy, mental slowing, anhedonia, and dysphoria [23]. IL-1, IL-6, and IFN-γ also induced anhedonia, despair, social withdrawal, and changes in sleep and learning patterns [9]. 

Inflammatory cytokines induce the enzyme indoleamine-2,3-dioxygenase. This enzyme catalyzes the synthesis of kynurenine from tryptophan, a well-known precursor of serotonin, melatonin, and nicotinamide [30]. The kynurenine pathway leads to the production of nicotinamide, thus, leading to the hypothesis that an over activation of this pathway could deplete tryptophan and sufficient serotonin synthesis might not occur [31]. Ruhe et al. reported that decreased levels of plasma L-tryptophan were associated with depression [32], while Maes et al. showed that decreased L-tryptophan levels were also associated with systemic inflammation [33]. Moreover, kynurenine was associated with depression-like symptoms in animal models, and the blockade of the enzyme inhibited depressive-like behavior in mice [34]. Additionally, kynurenine also metabolizes into quinolinic acid, which is an N-methyl-D-aspartate agonist, suggesting possible neuronal damage and other yet unknown cytotoxic pathways [35]. 

Cytokines also influence the dopamine synthesis pathway. In rat models, injection of IFN-α increased nitric oxide levels and decreased concentrations of dopamine and tetrahydrobiopterin (BH4), which is a cofactor for tyrosine hydroxylase, an enzyme that catalyzes the conversion of tyrosine into levodopa [36]. Interestingly, BH4 also plays a role in nitric oxide synthesis, suggesting that increased nitric oxide synthesis might limit the availability of BH4 for sufficient dopamine turnover [37]. 

Inflammation of the central nervous system can directly lead to certain psychiatric symptoms that develop into full-blown depressive or bipolar disorders [38]. Even when administered directly, cytokines affect the HPA axis and increase levels of the corticotropin-releasing hormone, adrenocorticotropic hormone, and the release of cortisol. These findings were reported to be present in patients with depression [39]. The relationship between depression and the levels of cytokines found in the cerebrospinal fluid (CSF) is also interesting. Kern et al. reported that dementia-free geriatric depression patients showed elevated levels of IL-6 and IL-8 in the CSF compared to healthy controls [14]. Miller et al. also reported that higher levels of CSF IL-1β, IL-23, and IL-33 were associated with increased odds of perinatal depression, whereas plasma cytokine levels did not show any significant associations [40].

Engler experimented further on this idea and administered healthy volunteers with small doses of endotoxin to stimulate the release of cytokines. He reported that a selective increase in IL-6 in the CSF leads to mood impairments, where higher levels are associated with greater deterioration [41]. Lindqvist et al. also reported that CSF levels of IL-6 were related to the severity of depressive symptoms in suicidal patients [42]. Contrarily, Carpenter reported that there were no differences in CSF levels of IL-6 in patients with unipolar major depression when compared to healthy controls. [43]. 

A more recent meta-analysis by Wang and Miller summarized the findings on CSF cytokine levels in various psychiatric disorders [44]. Patients with MDD showed a significant increase in IL-6 and IL-8, whereas levels of IL-1β and kynurenic acid were increased in patients with bipolar disorder. Patients with schizophrenia showed an increase in CSF IL-1β, IL-6, IL-8, kynurenine, and kynurenic acid.

Studies suggest that the relationship between the immune system and depressive disorders begins in the fetal period. Kowalczyk et al. explained that exposure to cytotoxicity during the prenatal period might affect the development of the immune system in favor of future depressive disorders [45]. Studies on patients with maternal depression have also confirmed that higher levels of maternal pro-inflammatory cytokines like IL-6 or TNF-α were associated with negative affect in infants [46]. Thus, it is possible that higher maternal inflammation levels contribute to higher rates of depression in children.

The impact of genetics and environmental factors on the relationship between cytokines and depression needs to be investigated. The heritability of cytokine production capacities was tested by de Craen et al. with ex vivo studies on twins and siblings, resulting in an estimated heritability of 53% to 86% [47]. Recent findings have suggested that certain genetic variants of cytokines are associated with clinical depression [48]. The NR3C1 gene, which is associated with polymorphisms of glucocorticoid receptors, is also potentially involved in the interaction of cytokines and depression. One study reported that variants of the gene were associated with susceptibility to depressive symptoms, but other studies failed to find such associations [49,50].

Stress is the most widely known environmental factor that affects the immune system, and there are various reports on stress contributing to elevated levels of inflammatory cytokines [51,52]. There is evidence that chronic stress such as marital distress or caregiving can increase levels of C-reactive protein (CRP) and other inflammatory markers [53,54]. In the presence of high-stress levels, the immune system becomes resistant to cortisol, a very potent anti-inflammatory endogenous agent. This is supported by a study on cancer caregivers who showed increased levels of inflammatory markers but decreased response elements for glucocorticoids despite similar cortisol levels [55]. 

### 2.2. Cytokines Changes and the Treatment of Depression

Cytokine levels are affected by antidepressant treatment, but the results are conflicting. Previous studies have shown that therapeutic doses of antidepressants—clomipramine, sertraline, escitalopram and trazodone lowered IFN-γ levels and increased levels of IL-10 [22,56], and clomipramine, imipramine, and citalopram decreased the levels of IL-1β, IL-6, TNF-α, IL-2, and IFN-γ [10].

A meta-analysis of various antidepressants showed that most of them, especially serotonin reuptake inhibitors, resulted in a decrease in IL-6 and IL-1β levels [11]. Kraus and Kast reported that treatment with mirtazapine induces an increase in plasma TNF-α level; however, Gupta et al. reported a decrease in the TNF-α level after the mirtazapine treatment [19,20,21]. Nevertheless, a meta-analysis by Kohler et al., including 45 studies, reported an overall decrease in IL-6, IL-10, and TNF-α levels after antidepressant treatment [15]. Munzer et al. also reported contradictory results in his study on the effect of antidepressants; citalopram increased the production of IL-1β, IL-17, and TNF-α; mirtazapine increased levels of IL-1β, TNF-α, and IL-22, but escitalopram decreased plasma levels of IL-17 [12]. A longitudinal study by Amitai et al. showed that antidepressant treatment lowers the level of plasma TNF-α in children and adolescents with depression. Similarly, Perez-Sanchez et al. reported an increase in TNF-α in youths with both first onset and recurrent depression, which subsequently decreased after antidepressant treatment [57,58]. A more up-to-date meta-analysis by Wiedlocha et al. stated that antidepressants significantly decrease plasma levels of IL-4, IL-6, IL-10, and IL-1β for serotonin reuptake inhibitors, but did not have significant associations with IL-2, TNF-α, and IFN-γ [13]. 

Besides antidepressants, antipsychotics and mood stabilizers are also often used, especially in cases of treatment-resistant depression. Among them, clozapine, olanzapine, lithium, and carbamazepine are associated with an increase in pro-inflammatory cytokines [59,60]. It is not yet clear whether this increase is due to the direct effect of drugs or due to the accompanying weight gain. Additionally, Jha et al. reported that higher baseline IL-17 levels were linked with greater therapeutic responses to antidepressant treatments, but there is not enough data to back this idea up [61].

It is no wonder that these studies have led to the trial of anti-cytokine agents for the treatment of depression. Maas et al. conducted a study using infliximab, a TNF-α antagonist, on elderly patients with depression. Although a few patients were treated effectively, the study was terminated prematurely because of deficient recruitment of patients [62]. Raison et al. reported that infliximab did not show enough efficacy as antidepressant medication, but hinted that baseline inflammatory status may be a determining factor for its effect [63]. In their cohort study, Wu et al. observed that patients with depression and psoriasis and those with psoriatic arthritis were treated with etanercept, adalimumab, and golimumab, which are also anti-TNF-α agents. Patients treated with anti-TNF-α agents tended to show a greater decline in clinical symptoms when compared to patients treated with other types of medication [64].

Increased levels of IL-6 are relatively common findings when looking at cytokine levels in patients with depression. Sirukumab, an IL-6 blocking agent, was reviewed for its possible effects on patients with psychiatric illnesses. Patients were likely to find relief from symptoms such as depression, rumination, or anhedonia. However, lack of hard data has been a stumbling block in the verification of the drug as a psychiatric medication. [65].

Da Silva et al. reported that psychodynamic psychotherapy could reduce plasma levels of pro-inflammatory cytokines in patients with depression [66]. Cognitive-behavioral therapy (CBT) is another type of therapy often used for patients with MDD. CBT for insomnia and pain resulted in decreased inflammatory markers in studies on patients with depression and rheumatoid arthritis [67,68,69]. Furthermore, some studies suggest that meditation and yoga may also reduce inflammatory responsiveness and have protective effects against depression [70,71].

The gut microbiome and the gut-brain axis, a bidirectional communication system between the gastrointestinal tract and the brain, have recently drawn attention in various medical specialties. Changes in the microbiome are known to have certain effects on inflammation, mood, and obesity [72]. Patients with depression were reported to have more antibodies against gut bacteria and higher intestinal permeability, sometimes called a “leaky gut,” allowing more movement of endotoxins inducing inflammations [73]. The gut microbiome also seems to be able to synthesize or mimic monoamines and catecholamines, thus, affecting the central nervous system (CNS) in various ways [74]. Despite the limited evidence, data suggests that probiotics may help to reduce depressive symptoms through anti-inflammatory effects, giving rise to novel treatment methods [75].

Studies focusing on lifestyle have shown that healthier diets are linked with lower risks of clinical depression, although conclusions should not be drawn too quickly due to difficulties in study design. A healthy diet is also known to decrease inflammatory markers. Several studies on Mediterranean diet styles have associated these with lower CRP and IL-6 levels [76,77]. A recent study suggested the possible antidepressant effects of the Mediterranean diet, as patients with depression who followed that particular type of diet showed no increases in IL-6 levels, whereas participants that did not follow a Mediterranean diet showed a larger increase [78]. Furthermore, a simple reduction in caloric intake also resulted in anti-inflammatory effects, and even antidepressant effects in rodent studies [79,80]. This anti-inflammatory effect is observable in intermittent fasting, short-time fasting, and time-restricted feeding situations [81,82]. 

A specific dietary component of interest is fish oil, or more specifically, its components: the omega-3 fatty acids, eicosapentaenoic acid and docosahexaenoic acid. Lower omega-3 fatty acid levels were associated with higher serum inflammatory marker levels in observational studies, but controlled trials did not produce significant changes in cytokine levels [83]. This might be a possible clue for using fish oil as a remedy for depression, but current meta-analyses have shown conflicting results; thus, hasty conclusions should be avoided [84,85].

### 2.3. Cytokines as Biomarkers of Depression

The search for a reliable diagnostic tool for depression is ongoing, as there are currently no concrete biomarkers that are universally applicable for the diagnosis of depressive disorders. As mentioned earlier, many studies have shown a possible link between cytokines and the clinical symptoms of depression, and it was only inevitable that researchers viewed cytokines as feasible biomarkers of the disease. A meta-analysis by Kohler et al. stated that plasma levels of IL-6, IL-10, IL-12, IL-13, and TNF-α were elevated in patients with depression [15].

Nevertheless, no large-scale study has yet proven that alleviation of depressive symptoms is linked to a direct decrease in plasma cytokine levels. Treatment with certain antidepressants indeed resulted in such decline, but it is not clear if the effects are pharmacologic or due to a change in clinical symptom severity [19]. 

Some findings suggest the possible use of cytokine levels to predict the prognosis of depression. Baune et al. reported a prospective study on elderly patients where IL-6 and IL-8 levels not only showed linear relationships with depressive symptoms but also baseline IL-8 levels were predictive of the first onset of mild to moderate symptoms within two years of follow-up [86]. A British cohort study also reported that baseline IL-6 and CRP levels predicted the development of cognitive and depressive symptoms in an average follow-up of 11.8 years [87]. 

## 3. General Considerations for Machine Learning in Neuroimmunology in Depressive Disorders 

Many studies have measured cytokine levels in depressive disorders, including meta-analyses. Although many research findings confirm the importance of neuroinflammation in the pathophysiology of depressive disorders, substantial heterogeneity has been observed. This might be due to both technical issues and clinical confounders. It is impossible to delineate a specific cytokine profile due to the disparate results, and this heterogeneity limits the potential for the casual use of cytokines as predictive measures in depressive disorders [5].

Accordingly, to obtain a more comprehensive picture of the inflammatory response system, it is necessary to consider anti-inflammatory cytokines and clinical variables in addition to pro-inflammatory cytokines. To implement the use of cytokines as biomarkers for depressive disorders, ML has been introduced. ML is useful for finding more complex nonlinear relationships between input variables (for example, cytokines and clinical characteristics such as age, sex, and depressive symptom severities) while examining multiple interrelated variables [88]. As neuroinflammation plays a role in the pathophysiology of depressive disorders, the ability to predict inflammatory biomarkers along with other clinical variables, such as the severity of depression, has been examined using ML. Ironside et al. investigated medical data from biobanks, including serum inflammatory acute phase reactants (CRP), in 12,589 participants. Excluding participants with any form of chronic inflammatory condition, the number of participants with lifetime MDD and without lifetime MDD decreased to 560 and 3588, respectively. Smoking history was available for 2809 participants, and the sample was further reduced and partitioned into training (60%, n-1687) and testing (40%, *n* = 1122) datasets. Logistic regression on the training dataset showed that a model including the count of CRP > 3 mg/L showed a trend to better predict probable MDD than a baseline model including only sex, lifetime smoking, and body mass index (*p* = 0.06). Subsequently, model estimates from the training set were used to predict values on the testing set. A classification rate of 84.5% was achieved by comparing the predicted target variable with the observed values. It showed that inflammation, as shown by the count of CRP > 3 mg/L, was a useful predictor of probable MDD. There were some limitations to this study. The relationship between chronic inflammation and MDD was not very strong because only the absolute elevation of CRP was a significant predictor and not the mean level. Due to the cross-sectional design, it was also not possible to suggest directionality between the two factors [89].

There are more in-depth studies investigating the relationship between cytokine-neuroimmunological biomarkers and depressive disorder using ML. We will further review the published material that has used ML with neuroimmunological data for the diagnosis of depressive disorders, and suggest further research directions

### 3.1. Differentiation of Unipolar Depressive Disorder from Bipolar Disorder Using Machine Learning

For clinical purposes, it is challenging to differentiate unipolar depression from bipolar depression. Although the division in diagnostic criteria seems to be well-defined, the definitive diagnosis of bipolar depression is not easy and can be confusing. In particular, the diagnostic conversion of unipolar depression later into bipolar depression has been reported to be up to 1.5% of patients per year [90]. The overall risk of conversion from initial unipolar depression to bipolar disorder (BD) in patients with unipolar depression participating in antidepressant trials has been reported to be 20.7% [91]. Accordingly, some studies have focused on the differentiation between these two types of depression for clinical purposes. There are currently no concrete biomarkers for the diagnosis of bipolar depression, as was the case with the diagnostic use of cytokines in depressive disorders. Moreover, there is no single definite biomarker or clinical characteristic that can distinguish BD from a depressive disorder. However, there are some commonly reported changes in cytokines shown in meta-analyses of BD patients when compared to healthy controls, such as IL-4, IL-6, IL-10, and TNF-α. These changes were also affected by clinical variables such as symptom severity, mood episodes, staging, and pharmacotherapy [92,93]. Interpretation of the results was limited by the heterogeneity of clinical variables and treatment settings between studies, insufficient standardization, and the lack of control for confounders in individual studies. Accordingly, it is not possible to identify definite biomarkers that are useful for the differentiation between depressive disorder and bipolar depression using traditional comparison methods. Therefore, we might consider introducing ML techniques for the interpretation of variables, including clinical parameters and neuroimmunological biomarkers, and reliable differentiation and diagnosis through pattern recognition of the data.

Wollenhaupt-Aguiar et al. used ML techniques for 54 outpatients with bipolar depression, 54 outpatients with unipolar depression, and 54 healthy controls, matched by sex and age. Patients were recruited from outpatient programs. The diagnosis of BD and unipolar depressive disorder was performed using the diagnostic criteria of the Structured Clinical Interview for DSM-IV-Axis I, Diagnostic and Statistical Manual of Mental Disorders-IV (SCID-I, DSM-IV). Cytokines, brain-derived neurotrophic factor (BDNF), thiobarbituric acid reactive substances (for the assessment of lipid peroxidation), and carbonyl groups (for the assessment of oxidative damage to proteins) were assayed. Accordingly, 10 serum biomarkers (IL-2, IL-4, IL-6, IL-10, TNF-α, IFN-γ, IL-17A, BDNF, thiobarbituric acid reactive substances, and carbonyl levels) were used as input variables in the model. Feature selection was performed using recursive feature elimination. The models were trained with a support vector machine algorithm with a linear kernel. For the comparison of bipolar depression versus unipolar depression, an area under the curve (AUC) of 0.69 with 0.62 sensitivity and 0.66 specificity using three selected biomarkers (IL-4, thiobarbituric acid reactive substances, and IL-10) was achieved. In addition, patients with unipolar depression and healthy controls also showed an AUC of 0.74, with 0.69 sensitivity and 0.70 specificity using seven variables (IL-6, carbonyl, BDNF, IL-10, IL-17A, IL-4, and TNF-α). [94]. However, all the patients in this study were under medication, and drug-free patients were not included, although there is a possible association between peripheral markers and pharmacotherapy.

Poletti et al. included 208 inpatients (127 patients with MDD and 81 patients with BD) and 32 HCs. Participants were 18–65 years old. They were diagnosed using SCID-I and DSM-IV. Cytokines (IL-1β, IL-1ra, IL-2, IL-4, IL-5, IL-6, IL-7, IL-8, IL-9, IL-10, IL-12, IL-13, IL-15, IL-16, IL-17, IFNγ, TNFα), macrophage migration inhibitory factor, chemokines (C-C motif ligand (CCL) 1, CCL2, CCL3, CCL4, CCL5, CCL8, CCL7, CCL11, CCL13, CCL15, CCL17, CCL19, CCL20, CCL21, CCL22, CCL23, CCL24, CCL25, CCL26, CCL27, C-X-C motif chemokine (CXCL) 1, CXCL2, CXCL5, CXCL6, CXCL8, CXCL9, CXCL10, CXCL11, CXCL12, CXCL13, CXCL16, C-X3-C Motif Chemokine Ligand (CX3CL) 1) and growth factors (fibroblast growth factor basic, granulocyte colony-stimulating factor, granulocyte-macrophage colony stimulating factor, platelet-derived growth factor-beta, vascular endothelial growth factor) were assayed. All analytes were entered as predictors into the elastic net models, including 5000 non-parametric bootstrapping procedures and inner and outer 10-fold nested cross-validation. For the comparison of bipolar depression vs. unipolar depression, it achieved an AUC of 0.97 with a balanced accuracy of 90%. In addition, unipolar depression versus healthy controls also showed an AUC of 0.99, with a balanced accuracy of 89%. The higher likelihood of MDD was associated with IL-1β, IL-2, IL-4, IL-6, IL-7, IL-10, IL-16, CCL20, CCL21, CXCL12, platelet-derived growth factor-BB (PDGF-BB), and vascular endothelial growth factor (VEGF). A higher likelihood of BD was also predicted by the levels of IL-9, TNFα, CCL3, CCL4, CCL5, CCL11, CCL25, CCL27, CXCL6, and CXCL11. Although both BD and MDD are associated with an overall increase in cytokine and chemokine levels, these research findings show that BD and MDD patients have different immune profiles and that these profiles can be used for differential diagnosis with high accuracy. Poletti et al. used a k-fold nested cross-validation procedure [95] when compared with the leave-one-out cross-validation in a previous study [94] to improve the model accuracy.

Zheng et al. compared 30 patients with MDD and 23 patients with depressive episodes of BD. The patients were aged 18–60 years and met the DSM-IV diagnostic criteria for MDD and BD. For feature selection, the demographic information, 24-item Hamilton Depression Scale (HAMD) score, Montgomery-Asberg Depression Rating Scale, Hamilton Anxiety Scale score, and neurotrophic factors (fibroblast growth factor-2 (FGF-2), nerve growth factor (NGF), insulin-like growth factor-1 (IGF-1), and VEGF) were used. Clinical symptom scale scores and blood tests were assessed at baseline and after eight weeks of personalized therapy, a stepwise discriminant analysis (forward stepwise method) was performed to select the variables. Using a model-based algorithm, they created a model with a fitting degree of 90.58% and an acceptable cross-validation error rate. The multivariate model based on age at onset, presence of family history, IGF at baseline, VEGF at baseline, delta for FGF-2, delta for NGF, and delta effect of HAMD factor 2 showed good performance. When only one type of variable was used, the AUC was relatively low (only sociodemographic and clinical characteristics: AUC 0.8007, only biomarkers: AUC 0.7725). The AUC increased to 0.9058 when both biomarker and sociodemographic/clinical characteristics were combined. The researchers included all of the discriminatory information in the preliminary feature selection rather than using the algorithms for variable selection, such as the previously mentioned support vector machine, to screen out statistically non-significant variables. The researchers also used 10-fold cross-validation and sequential forward selection procedure rather than a simple wrapper for the improvement of model accuracy [96].

### 3.2. Diagnosis of Depressive Disorder for Specific Populations Using Machine Learning

Walss-Bass et al. used data-driven exploration of depression and anxiety in adolescents [97]. A total of 254 adolescents aged 12–15 years were recruited. Thirty-nine cytokines and chemokines were assessed. In addition, 83 predictors (sex, race, time, 39 cytokines/chemokines, the interaction of each inflammatory marker with time, and random effects of participant ID and time) were included in the model for the prediction. In the longitudinal design, the component-wise gradient boosting algorithm was used to develop a predictive model for depression and anxiety in adolescents. Each model was reduced via backward elimination to maximize parsimony and generalizability. The reduced models showed that transforming growth factor-alpha (TGF-α) and female sex predicted higher levels of depression, while growth-regulated oncogenes predicted lower levels of depression. TGF-α is known to be a ligand that plays a role in regulating differentiation and proliferation in neurogenesis in the CNS [98]. TGF-α seems to influence depression in still developing adolescents. As immune responses between males and females vary [99], there seem to be differences in sex for the prediction capability of depression in adolescents [97].

In older adults, ML has also been applied for the diagnosis of depression. A total of 566 Alzheimer’s Disease Neuroimaging Initiative (ADNI) participants, including 396 with MCI, 112 with Alzheimer’s disease (AD), and 58 with normal cognition were assessed using the multi-analyte biochemical panel. They were assessed at baseline and 1-year follow-up. There were 146 plasma analytes on the panel. To assess the utility of immunoassays as biomarkers for depressive symptoms, researchers have applied an ML ensemble classification method. To assess the ability of sets of analytes to classify participants with or without depressive symptoms, random forest analyses were performed. The samples were classified into those with depressive symptoms (GDS ≥ 2), without depressive symptoms (GDS = 0), and buffers (GDS = 1). At baseline, the best set achieved an accuracy of 75.4% (sensitivity: 62.8%, specificity: 83.8%), and at 1-year, the accuracy increased to 80.9% (sensitivity: 59.5%, specificity: 94.2%). Extracted features included hepatocyte growth factor, insulin polypeptides, pregnancy-associated plasma protein-A, and VEGF [100]. In particular, insulin levels have been positively associated with depressive symptoms in this aged population. The mechanism of this association might be quite complex due to the role of insulin resistance in depression through dysregulation of the HPA axis and activation of insulin signaling by inflammatory cytokines [101].

## 4. Discussion

We reviewed the current knowledge of the relationship between neuroimmunology and MDD. Various cytokines, both pro-inflammatory and anti-inflammatory, are affected by depressive symptoms. As summarized in Table 1, patients with depression showed increased levels of IL-8, IL-10, IL-12, and IL-13, whereas IL-6 and TNF-α showed mixed results between studies. The direct administration of IL-1, IL-2, IL-6, TNF-α, and IFN-α was shown to induce depressive symptoms of clinical significance. Cytokine levels also responded to antidepressant medication, as in vivo levels of IL-2, IL-4, IL-10, and IL-6 decreased, and IL-22 levels increased after treatment. IL-1β, IL-17, TNF-α, and IFN-γ on the other hand showed varying results depending on the study. The induction of the kynurenine pathway of tryptophan by inflammatory cytokines is considered to be another possible contributing factor to depressive symptoms. Cytokines also affect the gut microbiome and are affected by lifestyle modifications such as a change in diet, resulting in various psychiatric symptomatic outcomes.

Knowledge of the role of neuroimmunological modulators in depression is still a growing field, as new methods are being developed to observe and measure both neurological changes and immunological changes in the human body. Further advances in neuroimaging, genetics, and molecular studies may lead to a deeper understanding of this topic. Nevertheless, advances in data gathering, sorting, and manipulation are already having a major breakthrough with the rise the ML and AI. 

We have reviewed studies on the use of ML using neuroimmunological biomarkers, including cytokines, in depressive disorders. We need to examine patients with a more integrative view. ML research focuses on pattern recognition rather than the simple peripheral level changes of some biomarkers. Although the number of participants was relatively small, the predictive value of the model was quite good. The consistency of results in the real-world is important for better predictive value. For clinical purposes, MDD has been primarily investigated among the various types of depressive disorders. In addition, elevated inflammation was a significant predictor of probable MDD classification [87]. The MDD group showed a higher probability of elevated inflammation than the non-MDD group. However, the cytokines to be used to predict depressive disorders remain unclear. Although there are some related cytokines, no known consensus exists for the use of specific neuroimmunological biomarkers for diagnosis due to the heterogeneity of research findings. A possible reason for the occurrence of this heterogeneity may be the different settings and the different clinical variables used for feature selection in the studies.

There are also many features extracted from ML studies. However, there is not much overlap among these features across different studies. To use these study results for actual clinical practice, specific features need to be replicated for reliability.

As researchers have more data for feature selection, including variable dimensions such as sociodemographic variables, clinical rating scale scores, and the levels of neuroimmunological biomarkers, higher accuracy can be achieved for the differentiation of depressive disorders from other clinical conditions. There are limitations to the actual clinical applicability of simply increasing the number of variables.

ML has mainly been applied for the diagnosis of depressive disorders. In addition, ML might be used for predicting other clinical variables such as therapeutic outcomes. Most of the studies conducted so far have cross-sectional designs. It is necessary to extend the use of ML to predict treatment outcomes through long-term follow-up studies.

## Figures and Tables

**Table 1:** Classification of cytokines and their roles in depression.

Type of Cytokine	Levels in Depressed Patients	Induces Depressive Symptoms	Response to Antidepressants
Pro-inflammatory			
IL-1	-	Yes [9]	-
IL-1β	-	-	Mixed results [10,11,12,13]
IL-8	Increased [14]	-	-
IL-12	Increased [15]	-	-
IL-17	-	-	Mixed results [12]
TNF-α	Mixed results [5,15]	Yes [16,17,18]	Mixed results [10,12,15,19,20,21]
IFN-γ	-	Yes [9]	Mixed results [10,13,22]
			
Anti-inflammatory			
IL-2	-	Yes [23]	Decrease [10,13]
IL-4	-	-	Decrease [13]
IL-10	Increased [15]	-	Decrease [13,15]
IL-13	Increased [15]	-	-
			
Mixed			
IL-6	Mixed results [5,15]	Yes [9]	Decrease [11,13,15]
IL-22	-	-	Increase [12]
IFN-α	-	Yes [23]	-

Abbreviations: IL: interleukin, IFN: interferon, TNF: tumor necrosis factor.

## Data Availability

Data sharing not applicable.

## Abbreviations

MDD: major depressive disorder; BBB: blood-brain barrier; IL: interleukins; IFN: interferons; TNF: tumor necrosis factor; ML: machine learning; AI: artificial intelligence; HPA: hypothalamic-pituitary-adrenal; BH4: tetrahydrobiopterin; CSF: cerebrospinal fluid; CRP: C-reactive protein; CBT: cognitive-behavioral therapy; CNS: central nervous system; BD: bipolar disorder; SCID: Structured Clinical Interview for DSM; DSM-IV: Diagnostic and Statistical Manual of Mental Disorders-IV; CCL: C-C motif ligand; CXCL: C-X-C motif chemokine ligand; C-X3-C motif chemokine ligand; AUC: area under the curve; PDGF-BB: platelet-derived growth factor-BB human; VEGF: vascular endothelial growth factor; FGF-2: fibroblast growth factor-2; NGF: nerve growth factor; IGF-1: insulin-like growth factor-1; HAMD: Hamilton Depression Scale score; TGF-alpha: transforming growth factor-alpha; ADNI: Alzheimer’s Disease Neuroimaging Initiative; MCI: mild cognitive impairment; AD: Alzheimer’s disease; GDS: the Geriatric Depression Scale
